# The development of an alcohol brief intervention e-coach for use by primary care clinicians

**DOI:** 10.1186/1940-0640-8-S1-A15

**Published:** 2013-09-04

**Authors:** Lauren M Broyles, Manu Singh, Keri Rodriguez, Susan Hayashi, Bob Monte, Adam Gordon

**Affiliations:** 1 VA Pittsburgh Healthcare System, Pittsburgh, PA, USA; 2 JBS International, Inc., North Bethesda, MD, USA

## Introduction

Rates of drinking, binge drinking, and driving under the influence of alcohol are higher among U.S. Veterans than non-Veterans, and approximately 20% to 30% of all U.S. Veterans screen positive for alcohol misuse ranging from risky drinking to alcohol dependence. To address these serious concerns, the U.S. Department of Veterans Affairs (VA) policies and clinical practice guidelines standardize expectations for alcohol SBI and provide clinical decision-making guidance. However, there are key provider-level barriers to BI uptake and implementation including (1) lack of alcohol BI knowledge and skills and (2) competing clinical priorities for limited available time. Rates of BI delivery by busy primary care clinicians are low, even when prompted by electronic reminders based in the VA’s electronic medical record. Currently, BI training and practical role supports are not systematically offered.

## Objective

The purpose of this project was to develop and assess user feedback on a computerized clinical decision support system (CCDSS) to assist primary care clinicians with the real-time delivery and documentation of alcohol BI delivery. We (1) systematically assessed primary care clinicians’ processes and preferences of alcohol-related care to inform the development of a CCDSS and (2) developed the BI E-Coach CCDSS and assessed its usability, feasibility, and acceptability among clinician stakeholders in primary care.

## Methods

We conducted on-site semi-structured interviews, observations, and surveys with key sets of primary care staff members before and after pilot testing the BI-E-Coach tool, to facilitate its development, and to assess its usability, feasibility, and acceptability.

## Results

E-coach prototype development is described in the contexts of primary care work flow, processes of alcohol-related care, and clinician preferences regarding the utility, feasibility, and acceptability of the BI-E-Coach. Additionally, initial implementation barriers, facilitators, and efficacy are reported.

## Discussion

Refinement of the E-coach, efficacy testing, and implications for implementation are discussed.

